# Spot-scanning proton therapy for early breast cancer in free breathing versus deep inspiration breath-hold

**DOI:** 10.2340/1651-226X.2024.28591

**Published:** 2024-02-26

**Authors:** Line Bjerregaard Stick, Louise Lærke Nielsen, Cecilia Bui Trinh, Ihsan Bahij, Maria Fuglsang Jensen, Camilla Jensenius Skovhus Kronborg, Stine Elleberg Petersen, Linh My Hoang Thai, May-Lin Martinsen, Helle Precht, Birgitte Vrou Offersen

**Affiliations:** a Danish Centre for Particle Therapy, Aarhus University Hospital, Aarhus, Denmark; b The Education of Radiography, University College Lillebælt, Svendborg, Denmark; c Health Sciences Research Center, University College Lillebælt, Svendborg, Denmark; d Institute of Regional Health Sciences, University of Southern Denmark, Odense, Denmark; e Department of Radiology, Lillebælt University Hospital of Southern Denmark, Kolding, Denmark; f Department of Experimental Clinical Oncology & Department of Oncology, Aarhus University Hospital, Aarhus, Denmark

**Keywords:** Breast cancer, proton therapy, deep inspiration breath-hold, comparative treatment planning, heart dose

## Abstract

**Background and purpose:**

Proton therapy for breast cancer is usually given in free breathing (FB). With the use of deep inspiration breath-hold (DIBH) technique, the location of the heart is displaced inferiorly, away from the internal mammary nodes and, thus, the dose to the heart can potentially be reduced. The aim of this study was to explore the potential benefit of proton therapy in DIBH compared to FB for highly selected patients to reduce exposure of the heart and other organs at risk. We aimed at creating proton plans with delivery times feasible with treatment in DIBH.

**Material and methods:**

Sixteen patients with left-sided breast cancer receiving loco-regional proton therapy were included. The FB and DIBH plans were created for each patient using spot-scanning proton therapy with 2–3 fields, robust and single field optimization. For the DIBH plans, minimum monitor unit per spot and spot spacing were increased to reduce treatment delivery time.

**Results:**

All plans complied with target coverage constraints. The median mean heart dose was statistically significant reduced from 1.1 to 0.6 Gy relative biological effectiveness (RBE) by applying DIBH. No statistical significant difference was seen for mean dose and V17Gy RBE to the ipsilateral lung. The median treatment delivery time for the DIBH plans was reduced by 27% compared to the FB plans without compromising the plan quality.

**Interpretation:**

The median absolute reduction in dose to the heart was limited. Proton treatment in DIBH may only be relevant for a subset of these patients with the largest reduction in heart exposure.

## Background

The newly published meta-analysis by the Early Breast Cancer Trialists’ Collaborative Group showed that regional lymph node irradiation including the internal mammary nodes (IMN) reduces the rate of recurrence, breast cancer mortality and overall survival for high-risk breast cancer patients [1–4]. Irradiation of IMN increases the complexity of the radiotherapy planning and can cause increased radiation exposure to heart and lung, leading to a risk of radiation-induced heart toxicity [5–8] and secondary lung cancer [9, 10].

Proton therapy in free breathing (FB) can reduce radiation dose to heart and lungs compared to state-of-the-art photon-based radiotherapy in deep inspiration breath-hold (DIBH) [11–13]. The potential, additional benefit of proton therapy in DIBH has been investigated in treatment planning studies concluding that DIBH does not add a relevant reduction of dose to the heart, since cardiac exposure is already minimized with proton therapy in FB [11, 12]. The Particle Therapy Cooperative Group Breast Cancer Subcommittee describes in a consensus statement that routine use of DIBH to reduce heart exposure in unselected patients does not appear beneficial, since little additional sparing of the heart is achieved unless significant inferior cardiac displacement [14]. When using *en face* fields, the separation of heart and chest wall in DIBH is only causing a small change in the water-equivalent path length to the heart due to the low-density of lung tissue [14]. We have observed radiation dose to the heart greater than expected for the patients that are highly selected in the Danish Breast Cancer Group (DBCG) Proton Trial [15, 16] when compared to the treatment planning studies. Thus, these selected patients may still have a relevant benefit from proton therapy in DIBH if the location of the heart is displaced inferiorly, away from the IMN.

The aim of this study is to compare proton therapy in FB versus DIBH for highly selected patients with left-sided breast cancer, exploring the potential benefit of introducing the DIBH technique to further reduce doses to the heart and other organs at risk (OAR). The delivery time for spot-scanning proton therapy can be considerable due to energy- and spot-switching times. Therefore, we aimed at creating proton therapy plans in DIBH with delivery times that are feasible with treatment in DIBH.

## Materials and methods

The first 37 patients with breast cancer referred from photon radiotherapy department Aarhus University Hospital and treated with proton therapy at Danish Centre for Particle Therapy (DCPT) were considered for inclusion. The patients were treated between 2019 and 2021. All patients were referred for proton therapy at DCPT based on having a mean heart dose (MHD) ≥ 4 Gy RBE or/and an ipsilateral lung V17Gy RBE ≥ 37% on an initial photon radiotherapy plan, fulfilling the criteria of the DBCG Proton Trial. The inclusion criteria for this study: left-sided irradiation, loco-regional irradiation including IMN, able to perform DIBH, clinical target volumes (CTVs) delineated on the planning CTs (Computed tomography), no previous radiotherapy in the chest area. The study was approved by the Aarhus University Hospital institutional review board.

### CT scans and delineations

All included patients had two radiotherapy planning CT scans performed: one CT scan in DIBH from the referring photon department and one CT scan in FB from the proton center. The CT scan in DIBH was performed with voluntary breath-hold using the Real-time Position Management (Varian Medical System). All patients were scanned in supine treatment positions on a breast board tilted by five degrees with one or both arms over the head. The head was rotated a bit to the opposite breast with the purpose to slightly move esophagus, trachea and thyroid gland away from the fields. The slice thickness was 3 mm for the CT scan in DIBH and 2 mm in FB.

The CTVs for the breast or the thoracic wall and nodes were delineated using the European Society for Therapeutic Radiology and Oncology consensus guidelines [17]. The delineated lymph node CTVs were level 1–4, the interpectoral nodes and the IMN. The heart was contoured according to the Danish Multidisciplinary Cancer Groups’ guideline [18].

Six patients had a bolus on the CT scan in DIBH. The bolus was delineated and assigned to air. The CT scans in DIBH contained metal markings of the scar or the breast. The Hounsfield units of these markings were not overwritten, since the markings created artefacts in the skin that made it difficult to ensure target coverage towards the skin.

### Proton therapy planning

Spot-scanning proton therapy plans in FB and DIBH were created for all patients for the purpose of the study. All plans had a prescribed dose of 40 Gy RBE in 15 fractions following the planning criteria by the DBCG guidelines [19] with RBE fixed to 1.1. Seven patients received level 1 irradiation, six patients received skin irradiation and one patient received simultaneous integrated boost, but for the purpose of this study, all patients were planned with level 1 lymph node irradiation, no boost and no skin irradiation. All plans were generated using Eclipse v16.1 (Varian Medical Systems) and optimized using single field optimization. The proton therapy plans were created with the isocenter located centrally relative to CTVs and using 2–3 fields with gantry angles ranging from 0 to 45 degrees. All fields were planned with a range shifter of 34 mm water-equivalent material and a snout position (distance between isocenter and snout) of 28 cm corresponding to a central axis air gap of 25.4–30.6 cm. All plans were optimized robustly using 14 worst-case scenarios: ±3.5% range uncertainty and ±5 mm setup uncertainty combined with ±3.5% range uncertainty. In the daily clinic, DCPT uses a fixed cutoff for minimum monitor units (MU) per spot of 2 MU. To reduce the delivery time for the proton plans in DIBH, the number of spots was reduced. It was done by increasing the minimum MU per spot from 2 to 5 MU and by increasing the spacing between the spots. The spot spacing for the FB plans was 0.35–0.425 times the full width at half maximum of the spot size in air. The spot spacing for the DIBH plans was fixed spacing of 0.9 cm. The used spot spacing for both the FB and the DIBH plans is used in daily clinic at DCPT. Treatment delivery time was estimated using an in-house simulator of pencil beam scanning delivery for a ProBeam (Varian Medical Systems) gantry [20].

### Plan evaluation

All proton plans were required to comply with the DBCG constraints for the nominal CTV coverage: 98% of the whole breast CTV or the chest wall CTV should be covered by 95% prescription dose and 98% of the lymph node CTVs should be covered by 90% of the prescription dose. Constraints for OARs followed the planning criteria by the DBCG guidelines [19].

For the robust evaluation, 14 worst-case scenarios were used: ± 3.5% range uncertainty and ± 5 mm setup uncertainty combined with ± 3.5% range uncertainty. The plans were required to comply with DCPT constraints for robust CTV coverage: 95% of the whole breast CTV or the chest wall CTV should receive at least 95% of the prescribed dose for all worst-case scenarios and 95% of the lymph node CTVs should receive at least 90% of the prescribed dose for all worst-case scenarios, but 1–2 worst-case scenarios below those constraints for lymph node CTVs could be accepted to limit dose to heart and lung [15]. OARs were not evaluated robustly following DCPTs breast cancer planning guideline.

Paired, two-tailed Wilcoxon signed rank test was used to statistically compare the proton plans in FB and DIBH. Pearson’s correlation coefficients were used to test the correlation between heart dose metrics versus inferior displacement of the heart. *P*-values ≤ 0.05 were considered statistically significant for both tests, MATLAB v2021b (MathWorks Inc.) was used for statistical analyses.

## Results

**Figure 1:**
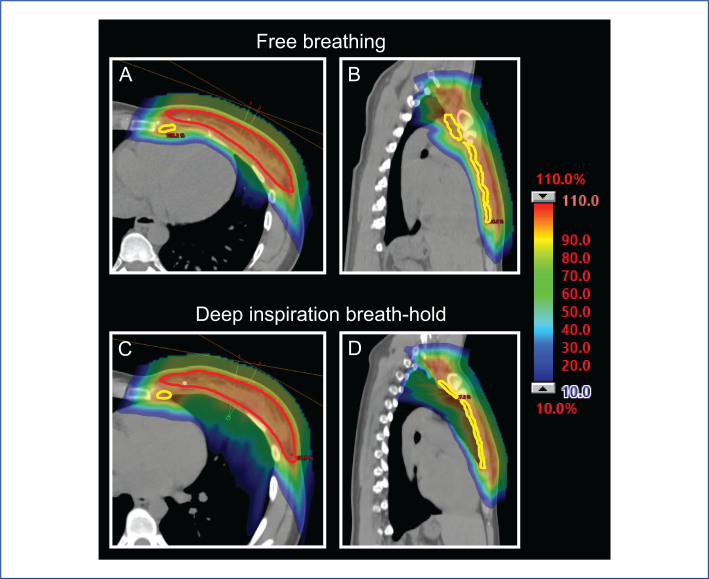
Patient example of dose distributions with proton therapy in free breathing (A, B) and deep inspiration breath-hold (C, D). Clinical target volumes for the breast (red structure) and for the internal mammary nodes (yellow structure) are delineated. The dose color wash shows dose ranging from 10% (blue) to 110% (red) of the prescription dose.

Sixteen of the 37 patients fulfilled the inclusion criteria and were included in the study (Figure S1 in Supplemental material). The patients’ median age was 55, ranging 28–74 years. Five patients were operated with mastectomy, 11 with breast conservation. All plans fulfilled nominal and robust target coverage evaluation. For the nominal evaluation, the median (range) CTVp V95% was 100% (99.5–100) for FB and 99.9% (99.1–100) for DIBH plans. All lymph nodes CTVn were covered with V90% = 100% for all plans. For the worst-case scenario robustness evaluation, the median (range) CTVp V95% was 99.2% (96.9–99.9) for FB and 98.5% (96.5–99.9) for DIBH plans. The median (range) IMN CTV V90% was 98.1% (95.2–99.8) for FB and 97.1% (92.8–99.7) for DIBH. A patient example of dose distributions can be seen in Figure 1.

**Figure 2:**
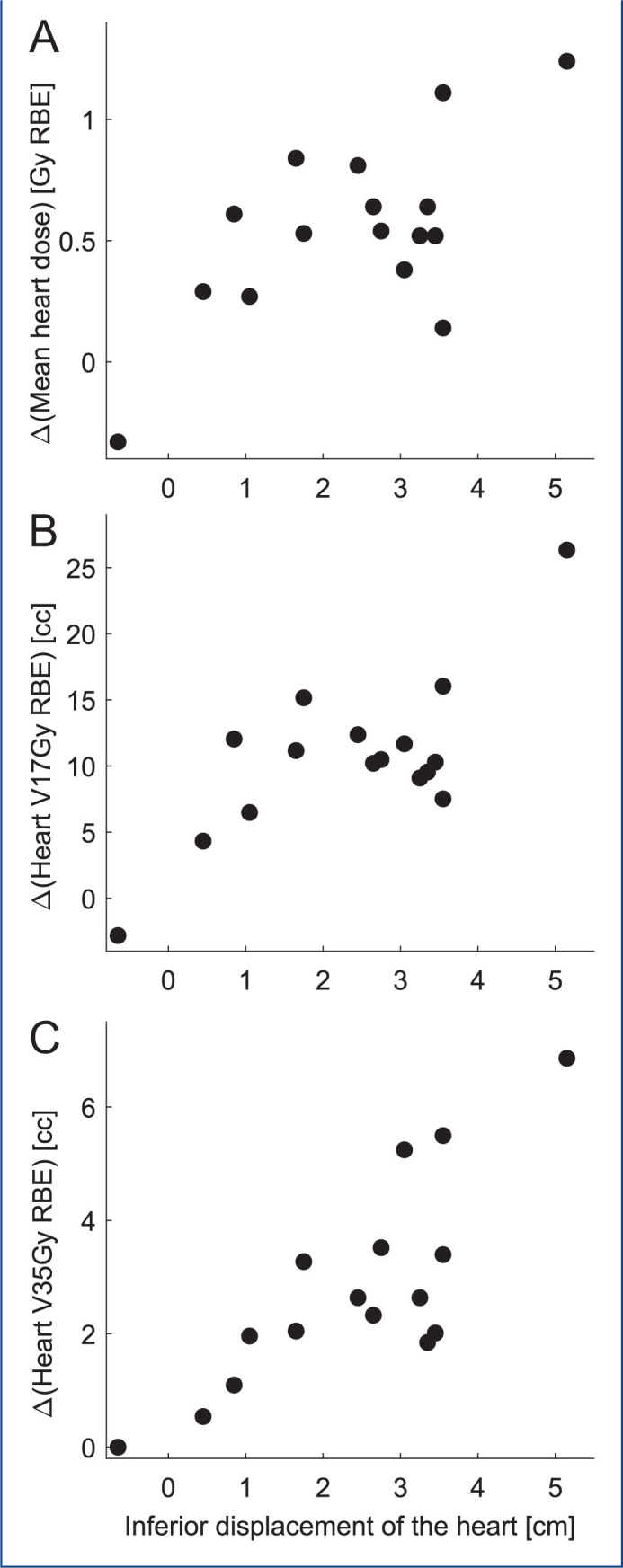
Differences in heart dose metrics as function of inferior displacement of the heart. The difference is defined as the metric in free breathing minus the metric in deep inspiration breath-hold. The Pearson’s correlation coefficients are 0.65 (*p* = 0.006) for mean heart dose (panel A), 0.73 (*p* = 0.001) for heart V17Gy RBE (panel B) and 0.80 (*p* = 0.0002) for V35Gy RBE (panel C). VXXGy RBE is the volume that receives XX Gy RBE or more.

There was a statistically significant difference in MHD between FB and DIBH technique: by applying DIBH, the median MHD was reduced by 47% (Table 1). Two patients had a MHD >1 Gy RBE reduction and nine patients had a MHD reduction 0.5–1 Gy RBE (Figure 2). The median heart V17Gy RBE was 15.1 cc in FB and 3.9 cc in DIBH and the median heart V35Gy RBE was 2.7 cc in FB and 0.1 cc in DIBH (Table 1). The median inferior displacement of the heart in DIBH was 2.7 cm (range, –0.7–5.2) which may explain the reduced dose to heart in the DIBH plans. As seen in Figure 2, there was a statistically significant correlation between the inferior displacement of the heart and mean heart dose (*r* = 0.65), heart V17Gy RBE (*r* = 0.73) and heart V35Gy RBE (*r* = 0.80). The maximum dose for esophagus was lowered statistically significantly from median of 33.1 Gy RBE in FB to median of 26.1 Gy RBE in DIBH. There was no significant difference for the other OAR dose metrics (Table 1).

**Table 1:** Dosimetric results for organs at risk with proton therapy in FB versus DIBH.

Dose metric	FB, median (range)	DIBH, median (range)	P
Heart, mean dose (Gy RBE)	1.1 (0.2–2.4)	0.6 (0.2–1.3)	0.0009*
Heart, V17Gy RBE (cc)	15.1 (1.1–36.7)	3.9 (0.3–19.9)	0.0005*
Heart, V35Gy RBE (cc)	2.7 (0.0–8.6)	0.1 (0.0–3.4)	0.00006*
Ipsilateral lung, mean dose (Gy RBE)	7.6 (5.8–9.8)	7.9 (6.9–10.6)	0.08
Ipsilateral lung, V17Gy RBE (%)	18.6 (14.4–24.1)	17.6 (14.8–25.6)	0.96
Esophagus, maximum dose (Gy RBE)	33.1 (24.5–35.7)	26.1 (10.4–33.0)	0.0009*
Trachea, maximum dose (Gy RBE)	28.3 (22.4–34.5)	26.9 (14.8–35.0)	0.41
Thyroid, mean dose (Gy RBE)	5.6 (1.2–12.9)	9.6 (0.6–14.5)	0.11
Humeral head, maximum dose (Gy RBE)	12 (8.8–17.1)	12.7 (7.1–18.3)	0.92
Skin, maximum dose (Gy RBE)	39.8 (37.9–42.4)	40.9 (39.1–42.4)	0.21
Combined CTVs, V107% (%)	0.2 (0–0.8)	0.1 (0–0.7)	0.53
Global maximum dose (Gy RBE)	109.4 (107.9–110.5)	109.7 (108.9–110.3)	0.30

VXXGy RBE is the volume that receives XX Gy RBE or more. FB: free breathing; DIBH: deep inspiration breath-hold; CTV: clinical target volumes.

The median (range) delivery time of fields was 323 seconds (270–570) in FB and 234 seconds (202–275) in DIBH, and the median reduction in delivery time was 27% for the DIBH plans compared to the FB plans (Table S1 in Supplemental material). The estimated number of breath-holds (assuming that patients perform breath-holds of 20 seconds) ranged from 11 to 15 breath-holds for the DIBH plans.

## Discussion

In this study, the proton therapy plans in DIBH were created with reduced delivery time compared to our standard clinical practice for plans in FB. The largest estimated number of breath-holds for the proton plans in DIBH was 15 breath-holds, which may be considered clinical feasible. To our knowledge, no other proton planning studies for breast cancer in DIBH have reported treatment delivery time. It was outside the scope of this study to examine the optimal trade-off between delivery time, plan quality and spot settings. All proton therapy plans met the target constraints; however, the proton planning in DIBH became more challenging than the proton planning in FB, and the coverage of the CTVs may have been slightly improved in the DIBH plans if reducing the treatment delivery time had not been considered or if the resolution of the CT scans in DIBH had been the same as for the CT scans in FB. There was more lung tissue in the proximity of the IMN CTV in DIBH compared to FB which made it more challenging to achieve robustness for IMN CTV in DIBH.

Treating patients with breast cancer in DIBH may induce underdosage of the target if the breath-holds are not stable and reproducible [21]. For voluntary breath-hold using an external marker block, the precision of the breath-holds may decrease when the block cannot be placed within the beam as the block will not necessarily be a good surrogate for the chest wall position. Another barrier for treatment in breath-hold is that pausing the image acquisition during cone-beam computed tomography (CBCT) is not possible for the ProBeam gantry-mounted CBCT system, and, therefore, it is not feasible to acquire CBCTs in DIBH. Also, for some proton therapy systems, there is a lack in integrating fluoroscopy during treatment which can be used to evaluate the intra-fractional reproducibility and stability of the breath-hold. The American Association of Physicists in Medicine (AAPM) Task Group Report 290 recommends that proton therapy manufacturers implement CBCT in breath-hold and real-time imaging of the patient [22]. Variations in intra- and inter-fractional motion in DIBH were not accounted for in the proton planning in this study. Proton therapy in FB appears robust against breathing motion when using an *en face* field arrangement and single field optimization [23, 24]. Breathing motion was not accounted for in the proton planning in FB.

Two comparative treatment planning studies found no statistically significant difference in MHD for proton therapy in FB versus DIBH. Ranger et al. included 14 patients with left-sided breast cancer and had an average MHD of 1.0 Gy RBE proton therapy in FB and 0.5 Gy RBE in DIBH [11]. Patel et al. planned 10 patients with left-sided breast cancer with an average MHD of 1.0 Gy RBE in FB and 0.7 Gy RBE in DIBH [12]. This current study included 16 highly selected patients treated with proton therapy due to challenging anatomies in respect to limiting heart exposure and found similar levels of heart exposure (a median MHD of 1.1 Gy RBE in FB and 0.6 Gy RBE in DIBH). Even though the median absolute dose reduction to the heart was limited, it could still be relevant to consider DIBH for a subset of these patients with the largest reduction in mean dose. Especially, since dose-response models for heart toxicity appear to have no lower threshold [5]. A large inferior displacement of the heart in DIBH does not necessarily imply a large reduction of dose to the heart compared with FB, and, thus, comparative proton treatment planning in FB and DIBH will be needed to identify patients with the largest dosimetric benefit.

## Supplementary Material

Spot-scanning proton therapy for early breast cancer in free breathing versus deep inspiration breath-hold

Spot-scanning proton therapy for early breast cancer in free breathing versus deep inspiration breath-hold

## Data Availability

The data that support the findings of this study are available from the corresponding author, LBS, upon reasonable request provided approval from relevant authority is available.
