# Supplementary material for: Spot-scanning proton therapy for early breast cancer in free breathing versus deep inspiration breath-hold
Source: Acta Oncol. 2024 Feb 26;63:28591. doi: 10.2340/1651-226X.2024.28591 (PMC11332550; doi:10.2340/1651-226X.2024.28591)
Supplement: Spot-scanning proton therapy for early breast cancer in free breathing versus deep inspiration breath-hold [file AO-63-28591-s1.pdf]

Supplementary material has been published as submitted. It has not been copyedited or typeset by Acta Oncologica.

Supplemental material

Spot-scanning proton therapy for early breast cancer in free breathing versus deep inspiration breath-hold

Line Bjerregaard Stick. Louise Lærke Nielsen, Cecilia Bui Trinh, Ihsan Bahij, Maria Fuglsang Jensen, Camilla Jensenius Skovhus Kronborg, Stine Elleberg Petersen, Linh My Hoang Thai, May-Lin Martinsen, Helle Precht, Birgitte Vrou Offersen

Table S1. Comparison of total CTV, number of treatment fields, total number of spots and delivery time for the proton plans in free breathing (FB) and deep inspiration breath-hold (DIBH). The p-values are from the paired, two-tailed Wilcoxon signed rank test.

**Patient**	**CTV (cm3)**		**Number of fields**		**Number of spots**		**Delivery time (s)**	
	**FB**	**DIBH**	**FB**	**DIBH**	**FB**	**DIBH**	**FB**	**DIBH**
**1**	395	455	2	2	9175	6516	274	202
**2**	502	542	2	2	10713	7043	288	210
**3**	1740	1723	3	3	27338	12831	465	275
**4**	473	486	2	2	10199	7648	322	225
**5**	392	405	2	2	10304	8126	324	259
**6**	742	685	2	2	16387	9696	392	233
**7**	415	437	2	2	10709	7576	319	232
**8**	662	680	2	2	12404	9151	339	256
**9**	2641	2267	3	2	35357	11760	570	250
**10**	440	458	2	2	9797	8325	330	261
**11**	252	291	2	2	7505	5631	270	202
**12**	497	573	2	2	10032	9111	299	237
**13**	1412	1466	3	2	25148	11343	419	234
**14**	538	668	2	2	10353	9303	301	236
**15**	492	481	2	2	9795	8375	325	221
**16**	295	326	2	2	6689	6133	313	232
**Median**	494	514	2	2	10329	8350	323	234
p	0.07		0.16		0.0004*		0.0004*	
