# Supplementary material for: Spot-scanning proton therapy for early breast cancer in free breathing versus deep inspiration breath-hold
Source: Acta Oncol. 2024 Feb 26;63:28591. doi: 10.2340/1651-226X.2024.28591 (PMC11332550; doi:10.2340/1651-226X.2024.28591)

Supplementary material has been published as submitted. It has not been copyedited or typeset by Acta Oncologica.

Supplemental material

Spot-scanning proton therapy for early breast cancer in free breathing versus deep inspiration breath-hold

Line Bjerregaard Stick. Louise Lærke Nielsen, Cecilia Bui Trinh, Ihsan Bahij, Maria Fuglsang Jensen, Camilla Jensenius Skovhus Kronborg, Stine Elleberg Petersen, Linh My Hoang Thai, May-Lin Martinsen, Helle Precht, Birgitte Vrou Offersen

Figure S1. Flowchart of the patient selection.

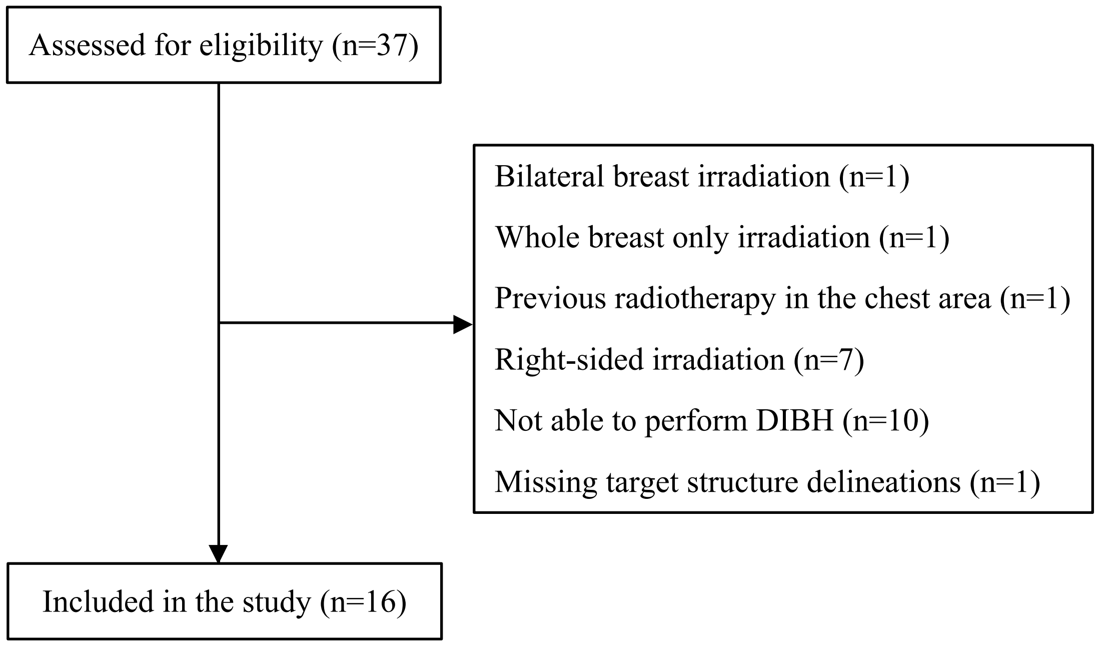

Supplement: Spot-scanning proton therapy for early breast cancer in free breathing versus deep inspiration breath-hold [file AO-63-28591-s2.pdf]
